# Review: Nutritional Needs of Honeybees and Legislation on Apiculture By-Products in Animal Nutrition

**DOI:** 10.3390/ani14152208

**Published:** 2024-07-30

**Authors:** Patrick Gernt, Julia Dittes, Ingrid Vervuert, Ilka U. Emmerich

**Affiliations:** 1 Faculty of Veterinary Medicine, Institute of Animal Nutrition, Nutrition Diseases and Dietetics, Leipzig University, 04103 Leipzig, Germany; p.gernt@web.de (P.G.); ingrid.vervuert@vetmed.uni-leipzig.de (I.V.); 2 Centre for Applied Training and Learning, Faculty of Veterinary Medicine, Leipzig University, 04103 Leipzig, Germany; julia.dittes@vetmed.uni-leipzig.de; 3 Faculty of Veterinary Medicine, Institute of Veterinary Anatomy, Histology and Embryology, Leipzig University, 04103 Leipzig, Germany; 4 Faculty of Veterinary Medicine, Institute of Pharmacology, Pharmacy and Toxicology, Leipzig University, 04103 Leipzig, Germany

**Keywords:** honeybee, pollen, royal jelly, sugar-enriched feedstuffs, supplements

## Abstract

**Simple Summary:**

Interest in beekeeping has significantly increased in recent years. A basic understanding of the nutritional and physiological requirements of honeybees is of great interest to improve their welfare and honey yields. The unique anatomic features of the gastrointestinal tract of bees, which enable bees to make optimal use of their food resources, are extensively described in this review. Nectar, pollen, and resins are raw materials for the synthesis of honey, beeswax, propolis, and royal jelly by the honeybee. All of these products are used for nutrition or as food supplements for animals. In the context of these products, the legal requirements for the use of apiculture by-products in animal nutrition are highlighted.

**Abstract:**

Honeybees are some of the smallest farmed animals, and apiculture by-products, e.g., honey, beeswax, propolis, royal jelly, and pollen contribute to animal nutrition. For the effective production of these by-products, the optimal development and nutrient supply of the honeybee is required. Beginning with the development of the mouth and anal pores on the second day of embryonic development, the digestive tract differentiates into the mouth and fore-, mid-, and hindgut during the pupal stage. The various glands within the oral cavity are particularly important, secreting enzymes and substances that are crucial for digestion and hive nutrition, e.g., invertase and royal jelly. Honeybees rely on a specialized caste system, with worker bees collecting nectar, pollen, water, and resin for the nutrition of the entire hive. Macronutrients, including proteins, carbohydrates, and lipids, obtained primarily from pollen and nectar, are essential for the growth and development of larvae and the overall health of the colony. Inadequate nutrient intake can lead to detrimental effects on larval development, prompting cannibalism within the hive. Apiculture by-products possess unique nutritional and therapeutic properties, leading to a growing interest in the use of honey, beeswax, propolis, and pollen as a feed additive. In recent years, the use of apicultural by-products in animal nutrition has been primarily limited to in vivo studies, which have demonstrated various positive impacts on the performance of farm animals. Honey, beeswax, propolis, royal jelly, and pollen are listed feed stuffs according to Regulation (EC) No. 68/2013. However, for animal nutrition there is not any specific legal definition for these products and no legal requirements regarding their ingredients as given for honey or beeswax in European food law.

## 1. Implications

As the main pollinators of crop plants, bees are of major interest for the production of agricultural products. Annually, EUR 153 billion are provided by insect pollination worldwide [1]. This amount represents a share of 9.6% of the total economic value related to the agricultural production of human food [1]. Therefore, it is of common interest that the nutritional needs of honeybees are well understood. Appropriately nourished bees are a basic element of a healthy and high-performance colony.

## 2. Introduction

In 2023, approximately 1,000,000 honeybee (*Apis mellifera* L.) colonies were kept in Germany. During this year, efficiently working bee colonies produced 33,761 t of honey in Germany [2]. In particular, honey is understood as one of the most effective natural products and is used, alongside royal jelly, propolis, pollen or beeswax, for numerous products in the pharmaceutical, cosmetic, food, and feed industries [3].

In contrast to the well-known requirements of farm animals, e.g. dairy cattle, pigs, or poultry, there is only limited knowledge of the honeybees’ nutrient demands [4]. In addition, the environment strongly impacts their foraging behavior [5]. Honeybees are confronted with negative influences such as agrochemicals and pathogens, as well as climate change [6].

Worker honeybees collect nectar and pollen to meet the colony’s nutritional needs. These resources are collected in surplus and preserved in the form of honey and bee bread to ensure an adequate supply of feed even during periods of scarcity. Large amounts of carbohydrates are contained in nectar and honey. Thus, nectar and honey are important at all stages and serve as high-quality energy suppliers. For brood rearing, growth, and social immunity, proteins and micronutrients are necessary, which are provided by pollen and bee bread [7].

In traditional medicine, the wound-healing properties of honey have been highly appreciated for centuries [8], and the health-promoting properties of bioactive substances from natural sources are of growing interest at present [9]. Consequently, scientific interest in investigating the effects of apiculture by-products as feed additives has increased significantly.

The aim of this review is to provide a scientific overview of the known nutritional needs of honeybees. In addition, bee products are characterized with regard to their value-determining ingredients and their use in animal nutrition. Legal requirements for the use of bee products as feed should be summarized.

## 3. Anatomy of the Digestive Tract of the Honeybee

The honeybee’s digestive tract consists of three main parts, the stomodeum, the mesenteron, and the proctodeum, which are not connected during the first stages of evolution [10]. The embryonic development of the honeybee’s digestive tract starts on the second day with the formation of the mouth and anal pores. On the third day, an intestinal tube is visible. In the embryonic phase, the stomodeum forms a circular valve and elongates to the cranial part of the mesenteron. In breaking through the wall, it forms the base of the later proventriculus and connects the first two parts. This situation enables the larva to take up food from the worker bees. The proctodeum remains closed until the end of the larval phase due to the prevention of larval food contamination [10]. During metamorphosis, new partitions arise between the stomodeum, ventriculus, and proctodeum. Extensive modifications then transform the simple gastrointestinal tract of the larva into the complex alimentary canal of the adult bee [11]. The stomodeum lengthens and transforms into the esophagus, crop, and proventriculus; the mesenteron changes into a looped cylindrical sac called the ventriculus; and the proctodeum differentiates into a thin anterior intestine and a large posterior intestine or rectum. The four larval Malpighian tubules dissolve, while around 64 new ones emerge around the pyloric region [12].

**Figure 1:**
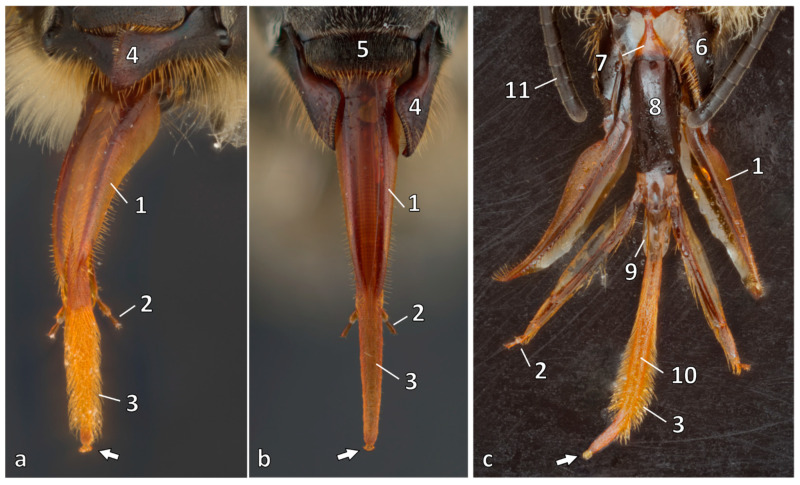
Proboscis of an adult worker bee: dorsal view of the dry (**a**) and nectar sticky (**b**) proboscis: galea (1), labial palpus (2), long hairy glossa (3) with labellum (arrow); also on view mandible (4) and labrum (5); ventral view (**c**) with parts artificially separated: additional: distal stipes (6), postmentum (7), prementum (8), paraglossa (9), salivary canal of glossa (10); also on view antenna (11). Source: preparation © Julia Dittes, photo © Jens Emmerich.

In the adult bee, food intake starts at the sucking apparatus, which is formed by the external mouthparts, among which the mandibles and the proboscis are considered. The mandibles, a pair of jaws originating from a reduced leg, are used by worker bees to chew pollen and to remodel wax for comb building [13]. Depending on the caste of the bee, there are differences in size, form, and hairiness. Two muscles, one abductor and one adductor, move the mandibles for different tasks. The proboscis consists of the two *maxillae* and the *labium*, simple structures forming a complex tubular system built to suck in fluids such as nectar and water [10]. Figure 1 shows an overview of the sucking apparatus. Each *maxilla* starts with a basal rod, called a *cardo*, which articulates at the proboscis fossa of the head proximally and with the *stipes* (Figure 1 (6)) distally. The *stipes* is a large component in the shape of a boat and ends with a small two-segment palpus. The main part of the sucking apparatus is the *galea* (Figure 1 (1)), a blade-like thin structure.

Between the *maxillae*, the *labium* begins with an elongated *prementum* (Figure 1 (8)) and a small triangular *postmentum* (Figure 1 (7)). Slender segmented palpi attach to the *prementum* with a basal lobe following four segments (Figure 1 (2)).

Another component is the lingular lobe, with two *glossae* forming the tongue (Figure 1 (3)) and a pair of short *paraglossae* (Figure 1 (9)). The long and hairy tongue consists of membranized and sclerotized bands, is oval in the transverse section, and ends with a spoon-shaped lobe, the so-called *labellum* (Figure 1 (arrow)). Important for the tongue mechanism, an elastic rod is present [14] in the inner wall, as well as a median groove that widens into a channel, the salivary canal of the tongue (Figure 1 (10)).

When a bee starts to take up liquid, the proboscis unfolds, with the galeae forming a roof and the palpi and marginal hairs closing it posteriorly. Whereas the base of the glossae is enclosed, the rest is freely moveable. The tongue is dipped into the fluid. In the moment that it extends to its full length, all of the hairs are erected simultaneously and extend across the surface. With the contraction of the tongue, as well as the cibarial pump, the nectar, for example, is sucked into the mouth [14].

In the honeybee’s oral cavity, there are different secretory glands fulfilling important tasks for the beehive’s nutrition. The hypopharyngeal glands are paired anatomical structures located in the head [15]. These glands have an age-specific function. In young nurse bees, they secrete the protein of royal jelly. After 12 days, the secretion changes to enzymes involved in honey production and preservation, such as invertase, which is necessary to hydrolyze sucrose with the help of alpha-glucosidase [16]. Besides the hypopharyngeal glands, there are the mandibular glands, which produce fatty acids for royal jelly and the basic components of the queen pheromone and alarm pheromones of honeybee workers [17].

**Figure 2:**
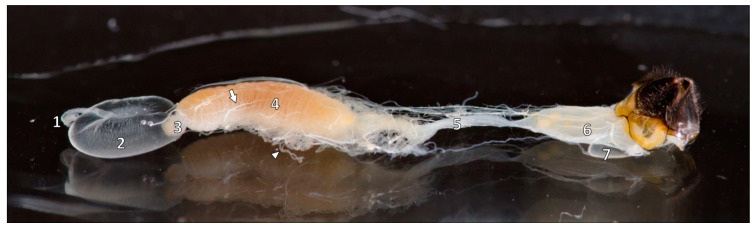
The isolated digestive tract of an adult worker bee: esophagus (1), crop/ingluvies (honey stomach) (2), proventriculus (3), ventriculus (4), proctodaeum with anterior intestine (colon/small intestine) (5) and posterior intestine (rectum) (6); also on view venom sac (7), tracheae (arrow) and Malpighian tubules (arrowhead). Source: preparation Ilka Emmerich, photo © Jens Emmerich.

**Figure 3:**
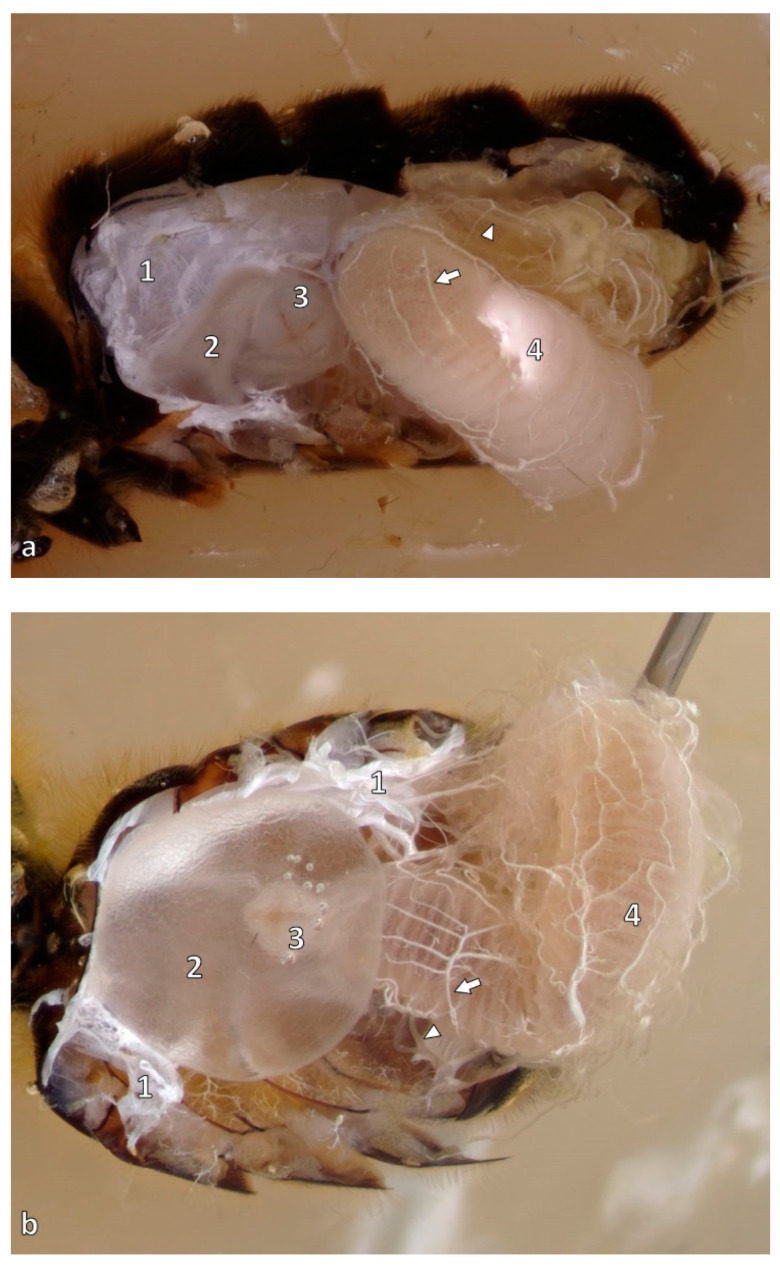
Lateral (**a**) and dorsal (**b**) view of the abdomen of an adult worker bee: tracheal sacs (1), crop/ingluvies (honey stomach) (2), proventriculus (3), ventriculus (4), also on view tracheae (arrow) and Malpighian tubules (arrowhead). Source: preparation Julia Dittes, photo © Jens Emmerich.

After passing through the mouth, the food enters the foregut, being transported through the esophagus (Figure 2, (1)), which enlarges into the crop, commonly known as the honey stomach (Figure 2 and Figure 3, (2)). As this organ is not functionally a real stomach, the term crop or *ingluvies* is used. The crop enables the honeybees to carry resources to and from the hive [13]. With the circularly folded wall allowing enormous dilatation, approximately 70 mg of nectar can be stored in it [18]. The foregut is separated from the ventriculus by the proventricular valve to prevent untimely digestion [13]. This is an X-shaped opening formed by four triangular folds with groups of spines on their upper sides (Figure 2 and Figure 3, (3)). On the inside of each fold, longitudinal and circular muscle fibers surround the whole proventriculus. The *ingluvies* is prolonged as a thin double-walled tube in the ventriculus, completing the valve system [10].

**Figure 4:**
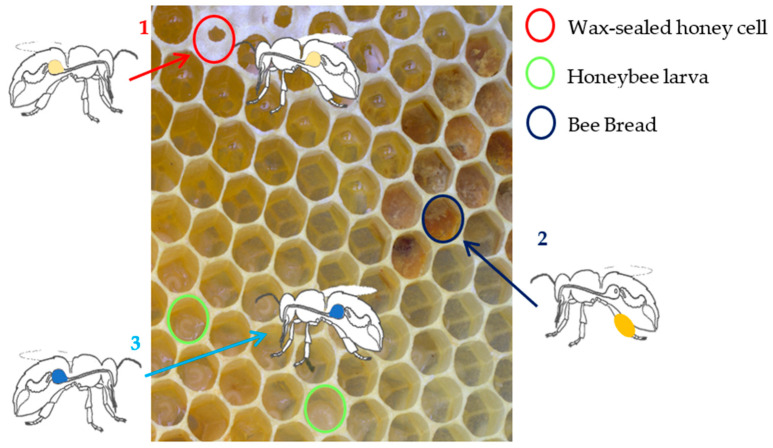
Schematic presentation of the working sequence during food collection in the beehive. Adapted from Wright et al. [24]. (1) In their honey stomachs, foragers transport nectar back to the hive. The nectar is transferred by trophallaxis. Storer honeybees depose it into cells where nectar ripens into honey and is later sealed with wax. (2) Foragers transport collected pollen back to the hive. Foragers add nectar to form pollen pellets which are deposed to cells next to the brood area. There, the pellets ferment to bee bread. (3) Water is mainly stored in the crop of foragers or transferred by trophallaxis into the crop of storer honeybees. Occasionally, storers depose water into cells. Source: © Ilka Emmerich.

The midgut or *ventriculus* is the physiological stomach of the bee. Forming the largest part of the gastrointestinal tract, the cylindrical tube lies in a U-shaped loop in the left part of the abdomen (Figure 2 and Figure 3, (4)). The beginning of the midgut is formed by two blind sacs, being of major importance for the resorption of water and ions. Moreover, the ventriculus secretes enzymes and resorbs nutrients, which are transported through the hemolymph [19]. Two to three muscle layers of longitudinal and circular fibers ensure the food transport of, e.g., pollen grains [19,20].

Food particles then exhibit a direct passage from the ventriculus to the anterior intestine, without an interposed valve. A wider pylorus region opens up into the small intestine, a small, looped tube with six longitudinal epithelial folds and a circular muscle layer (Figure 2, (5)). In the pylorus, the Malpighian tubules, an appendage of the alimentary canal with eliminative renal-like functions, end (Figure 2 and Figure 3, (arrowhead)). A sac-like structure called the posterior intestine or rectum (Figure 2, (6)) terminates the gastrointestinal tract. Its thin walls and numerous folds allow dilatation and, likewise, the storage of feces. Due to the extensibility of the rectum, honeybees do not eliminate their feces before they first leave the hive at the age of three weeks [18].

## 4. Macronutrients

### 4.1. Food Collection

Honeybees live together in a highly specialized social fabric in which their tasks are clearly distributed. A caste of sterile female workers assumes responsibility for the collection of food [21]. These foragers collect nectar, pollen, water, and tree resin to transport them back to the beehive, as shown in Figure 4 [22].

In the beehive, foragers transfer nectar to storer bees, which deposit it into the cells. There, the nectar ripens into honey. The pollen is, in turn, directly deposed by the foragers into cells next to the brood area after being formed into pollen pellets, to which saliva is added. Thus, bee bread is formed. The highly specialized nurse bees are the main consumers of bee bread, which they require to biosynthesize jelly in their enlarged food glands. By trophallaxis, they share the jelly with the larvae, other nurses, foragers, drones, and the queen [23].

### 4.2. Protein

The natural source of protein for honeybees is pollen. It is collected by the bees from blossoms as needed and passed into the colony. However, there is wide variation in the protein content of pollen (Table 1), ranging from 2.5% to 61% of dry matter (DM) [24]. The protein levels in pollen depend on the season and the geographic location [25]. In particular, bee-pollinated plants such as fruit trees, clovers, and alfalfa contain high levels of pollen protein [26].

In the honeybee’s midgut, the ingested proteins are digested and degraded enzymatically into peptides and amino acids [26]. Subsequently, these molecules are absorbed by the midgut’s cells and are available for metabolism in growing tissues, secreting glands, and muscles [28].

From the proteins obtained from pollen, nurse bees biosynthesize a high-protein secretion for their brood. From the fourth day, worker-destinated larvae are fed with worker jelly, whereas queen-destinated larvae receive the higher-grade royal jelly throughout the entire development period [29]. Approximately 25 to 37.5 mg of protein is needed to supply the needs of a single larva during its rearing period [30]. Consequently, these amounts of protein enable the honeybee larva to grow a hundredfold within the first 5 days [31]. Filipiak considered the botanical origin of pollen, the geographical location of the region where pollen was collected, and the differences between male and female larvae when examining the factors that might affect the nutrient composition of the larvae’s diets. Redundancy analysis revealed that the primary determinant of nutrient composition was the botanical origin of the pollen, explaining 70.2% of the variation. The location accounted for 20.2%, while the sex of the larvae contributed 8.43% [32].

If there is a qualitative or quantitative lack of protein, honeybees start to cannibalize their brood in order to ensure sufficient nutrition of the remaining larvae. The young larvae are killed, and the protein is used to feed other larvae [33]. Thereby, honeybees attempt to prevent their larvae’s malnourishment, leading to physical and cognitive malformation. Malnourished larvae have a diminished body size and weight [34], less developed ovaries [35], and decreased longevity and worse performance in terms of brood-related behaviors [36]. As a consequence, a whole population is affected negatively if there is a nutritional imbalance within the juveniles’ diet [37].

Adult honeybees ensure their protein intake in the form of bee bread (ambrosia), which is a mixture of regurgitated nectar, honey, and glandular secretions [38]. Due to microbial fermentation, ambrosia has increased bioavailability, since the walls of pollen are fermented and enable the facilitated assimilation of the pollen’s nutritionally valuable content [39].

Furthermore, honeybees have the ability to enrich proteins in their hemolymph. The storage of proteins enables the honeybee to compensate for long periods of restricted protein intake. The main storage protein is vitellogenin, which is also a precursor for numerous other proteins. Consequently, this lipoprotein is the basis of successful overwintering and is furthermore of great significance for the onset of foraging by workers and the honeybee’s longevity via the binding of pathogens [40].

### 4.3. Mono- and Oligosaccharides

Honeybees use nectar and honeydew as their natural sources of mono- and oligosaccharides, such as glucose, fructose, and others. Approximately 11 mg of mono- and oligosaccharides per day is necessary for a worker bee, and a colony of 50,000 honeybees needs approximately 318 kg of mono- and oligosaccharides (expressed as DM) annually [41]. During the 5-day development of the larvae, each of them needs approximately 59.4 mg of simple sugars. Moreover, on the last 2 days, the sugar content of the brood feed increases from 18% to 45% [42].

Collected and transported by foragers to the hive, nectar is stored in beeswax-sealed cells as honey. During the returning flight, the transformation from nectar to honey starts in the bee’s honey stomach [43]. In the hive, invertase and diastase, as well as glucose oxidase, are excreted and water is reduced to convert nectar into honey [44]. On average, honey is composed of 38% fructose and 31% glucose and some di- and trisaccharides [45]. Due to their low glycogen stores, adult worker bees are highly dependent on these colony food stores [30] to fulfil their social obligations.

An insufficient supply of mono- and oligosaccharides is often a problem in spring, when nectar sources are still rather limited, and the winter stores are already exhausted. Under these circumstances, the number of reared larvae decreases [46].

### 4.4. Lipids

Pollen is the honeybee’s main source of lipids [47]. The pollen’s lipid content varies between 0.8% and 18.9% [48]. It was shown that lipids increase pollen’s attractiveness among honeybees [49]. Especially during the brood stage, lipids are metabolized and are considered important energy suppliers [50].

Moreover, pollen contains sterols, which are essential to honeybees. Workers have the ability to convert phytosterols into the bee’s major sterol, 24-methylenecholesterol [51]. This sterol is the main precursor for essential hormones, e.g., the molting hormone, which regulates the honeybee’s growth [47]. Furthermore, it was shown that the addition of 0.1% cholesterol or 24-methylenecholesterol to the honeybee’s diet positively influenced the bee’s survival and brood production [52]. Worker bees have the unique ability to transfer sterols to the larvae through the brood’s food. This process is independent of the sterol level in the honeybee’s feed and enables consistent levels of 24-methylenecholesterol in the offspring [53].

## 5. Legal Requirements

Legally, apiculture by-products (honey, beeswax, propolis, royal jelly, pollen) are listed as feed materials in accordance with Part C of the Annex to Regulation (EU) 68/2013, meaning that these products and products derived from them can be processed or used in their natural state in animal feed. This regulation also specifies the total sugar content calculated as sucrose as a mandatory labeling element [54]. In addition, as for any feed, general requirements for safety and placing on the market in accordance with Article 4 of Regulation (EC) No. 767/2009 must be complied with [55].

For feed supplemented with bee products and intended for non-food-producing animals, Article 16 of Regulation (EC) No. 178/2002 also applies, which prohibits the labeling, advertising, or presentation of feed that may mislead the consumer [56].

Profound legal definitions for apiculture by-products can currently only be derived from European food law for honey and beeswax. In Annex I of the EU-Directive 2001/110/EC, honey is defined as a natural sweet substance produced by *Apis mellifera* L. from the nectar of plants or from secretions of living parts of plants or excretions of plant-sucking insects on the living parts of plants, which the bees collect, transform by combining with specific substances of their own deposit, dehydrate, store, and leave in honeycombs to ripen and mature. In addition, different types of honey (e.g., comb honey, extracted honey, filtered honey) are defined and composition criteria for honey (e.g., sugar content, moisture content, maximum hydromethylfurfural content) are specified in this regulation [57].

As wax, obtained by melting the walls of the honeycomb made by the honeybee, *Apis mellifera* L., with hot water and removed of foreign matter, yellow beeswax is defined in the Commission Regulation (EU) No. 231/2012. White beeswax is obtained by bleaching yellow beeswax and is consequently a synonym for yellow beeswax. Moreover, the morphology of beeswax is described in more detail as yellowish with yellowish to greyish pieces or plates with a fine-grained and non-crystalline fracture, having an agreeable, honey-like odor. With regard to the purity of beeswax, specific information on e.g., the acid value, the peroxide value and the maximum residue limits for arsenic, lead and mercury is provided by the Commission Regulation (EU) No. 231/2012 [58].

## 6. Honey

Honey is a natural product, and its physical characteristics, e.g., color, smell, taste, or consistency, are strongly affected by the geographical landscape as well as the climate conditions of the region where the nectar and honeydew are collected by the honeybees. More than 300 bioactive ingredients have been found in honey, although fructose and glucose account for 95–99% of honey’s dry matter [59]. However, even small amounts of proteins (0.2–1.6%) are detected which are attributed to secretions of the honeybee’s salivary glands but mainly source from pollen protein [60]. Moreover, approximately 0.57% of organic acids are found in honey, which are formed by sugars that are decomposed by enzymes of the honeybee actually serving to ripen the honey [61]. Thanks to the low pH of honey, even vitamins are preserved. In addition to vitamin C, especially the vitamins of the B-complex, e.g., thiamine (B1), riboflavin (B2), nicotinic acid (B3), pantothenic acid (B5), pyridoxine (B6), biotin (B8), and folic acid, are included [62]. Phenolic compounds in honey (e.g., flavonoids, hydroxybenzoic acids) did arouse scientific attention because of their antioxidant activity and the fact that volatile organic compounds from the nectar provide the characteristic smell of unifloral honeys [60]. The mineral content of honey ranges from 0.04% to 0.2% including macro- and microelement minerals. This chemical group is represented in honey by, e.g., potassium, magnesium, calcium, iron, phosphorus, sodium, manganese, zinc, and silver [63].

Depending on the climate conditions, honey contains 15–17% water, making it the second main ingredient [64]. The moisture content in honey is a major limitation for its stability and negatively affects its resistance against microbial spoilage during storage [65]. Therefore, it is legally required that the moisture content is, in general, no higher than 20% [57].

Hegazi et al. [66] studied the effects of using honey as a feed additive in drinking water for broiler chicks. Therefore, a total of 120 one-day-old broiler chicks were randomly divided into four groups: (I) control group, (II) honey control group, (III) control vaccinated group, (IV) honey vaccinated group. The study lasted 28 days and all groups were fed equal diets. Groups (II) and (IV) received a solution of 10% coriander honey for drinking during the entire study period. Vaccination against Newcastle disease (ND) was given to group (III) and (IV) on day 5 by ocular route and on day 14 in drinking water. At 7, 14, 21, and 28 days of age, body weight was evaluated, and five chicks were slaughtered for taking serum samples (for ND antibody titers). In addition, lymphoid organs (bursa of Fabricius, thymus, and spleen) were removed to determine their weight. Mortality was monitored during the whole trial [66].

It was found that body weight was significantly (*p* < 0.05) increased in the honey-supplemented groups during the last 2 weeks of the experiment. Moreover, the relative weights of the lymphoid organs were increased at all periods in group (II) and (IV) (*p* < 0.05). The highest ND-antibody titers were always found in group (IV) (*p* < 0.05), which was additionally the only group without any deaths. As a conclusion, Hegazi et al. assume an antimicrobial and immunostimulant activity of honey, opening perspectives for the use of honey as a food additive to improve chickens’ performance [66].

## 7. Beeswax

Beeswax has multiple uses in the beehive. Honeybees secrete wax to build honeycombs, and this enables them to store honey as an energy source [67]. Due to its hydrophobic properties, beeswax protects the hive against humidity and consequently positively affects the hive’s thermoregulation [68]. Moreover, it is postulated that beeswax has antimicrobial properties, and it has been shown that the wax exerts inhibitory effects on the growth of pathogens, e.g., *Staphylococcus aureus, Salmonella enterica*, or *Candida albicans* [67].

In order to use beeswax for human or animal nutrition, the combs are melted, and the wax is subsequently extracted, cleaned, and purified [68]. More than 300 different substances are found in beeswax. However, this wax consists of 67% esters of higher fatty acids and alcohols [69]. Small quantities of hydrocarbons and acids are also found in beeswax. The typical smell and taste of beeswax is determined by approximately 50 aroma components [69].

Gaafar et al. [70] studied the influence of beeswax-supplemented diets on the performance of sheep. Therefore, 18 male 5-month-old Assaf lambs were divided into three equal groups which were fed the following ratios for 90 days: (I) control group, (II) supplementation of 2 g beeswax/animal/day, (III), supplementation of 4 g beeswax/animal/day. The lambs were weighed biweekly before feeding in the morning. In order to calculate the feed digestibility, fecal samples were taken twice a day for a total of 5 days. Rumen liquor and blood serum were sampled for further analyses.

Based on the study results, Gaafar et al. [70] concluded that the supplementation of 2 or 4 g beeswax/animal/day increases growth performance by increasing the feed intake and digestibility of nutrients. Moreover, rumen fermentation and serum metabolites were increased, leading to a higher economic efficiency. The results show that the average daily gain increased by 16.8% (II) and 35.8% (III) compared to the control group [70].

## 8. Propolis

Propolis is a mixture of approximately 50% resin, 30% wax, 10% essential oils, 5% pollen, and 5% other substances [71], including amino acids, minerals, sugars, vitamins B, C, and E, flavonoids, and aromatic compounds [72]. Depending on the plant source, the chemical composition of propolis differs, but it is mainly dominated by, e.g., aromatic acids, terpenoids, hydrocarbons, fatty acids, and alcohols [73]. Propolis plays an important role in social immunity in the beehive [74]. The antimicrobial properties of the resin protect the hive against microbial infections, and cracks and crevices in the hive are sealed by this sticky substance [75].

The flavonoid components of propolis are considered to influence the bacterial metabolism and thus contribute to the antimicrobial action of propolis [76]. Thereby, the flavonoid content of propolis is strongly affected by its geographical origin. Woźniak et al. [77] detected the flavonoid concentrations ranging from 3.1 to 46.1 mg/g propolis just in 15 different regions of Poland.

An in vivo study by Klaric et al. [78] demonstrated the protective effects on the livers of broiler chickens through the dietary supplementation of propolis. A total of 200 1-day-old un-sexed Ross 308 chickens were divided into four groups. The four feeding groups were supplemented with different levels of propolis and bee pollen per kg diet over 42 days, as follows: (1) 0.25 g of propolis and 20 g of bee pollen; (2) 0.5 g propolis; (3) 1.0 g propolis; (4) 20 g bee pollen. The control group was not fed any supplements. At the end of the fattening process, 10 broilers of each group were randomly slaughtered to obtain liver samples for a necropsy examination. The histological analysis of the liver tissue showed that the dietary supplementation of propolis and bee pollen reduced various forms of regressive liver lesions, e.g., vacuolic degeneration, steatosis, and the necrosis of the parenchyma. Additionally, pathological findings in the liver tissue, e.g., clusters of lymphocytes among the hepatocytes and bile ductuli hyperplasia, were diminished. The extent of the pathological findings was described with labels ranging from “does not exist” to “extremely strongly expressed” [78].

## 9. Royal Jelly

Produced in the hypopharyngeal (protein part [18]) and mandibular (fatty acids part [16]) glands of worker bees, royal jelly ensures the nutrition of the larvae and the queen [79]. Royal jelly is composed of approximately 50% protein [80], 30% carbohydrates [81], and 3–6% lipids (expressed in DM) [82].

The positive impact of using royal jelly as a feed ingredient on the performance of quails was investigated by Seven et al. [83]. A total of 216 43-day-old Japanese quails (*Cortunix cortunix japonica*) were divided into three groups. The control group was fed a basal diet, group 2 a diet supplemented with 4 g propolis/kg diet, and group 3 a diet supplemented with 500 mg royal jelly/kg diet. The quails’ performance was observed for 74 days through the daily collection and weighing of eggs and the determination of the feed intake and feed conversation ratio. Furthermore, 100 eggs from each group were incubated, and unhatched eggs were cracked to classify them as infertile or as exhibiting embryonic death. At the end of the investigation period, 10 hens from each group were slaughtered to collect blood samples and liver tissue to determine the lipid peroxidation and antioxidant enzyme activity. Seven et al. [83] determined that the shell weight, shell thickness, and shell rate were improved by the dietary supplementation of propolis and royal jelly (*p* < 0.05), whereas the hatchability was not affected (*p* > 0.05). In addition, the glutathione (*p* = 0.012) concentration and glutathione peroxidase (*p* = 0.018) activity were significantly higher in the royal jelly group [83].

## 10. Pollen

Pollen is collected by honeybees from plant anthers and afterwards mixed up with up to 10% of secretions from their salivary glands or nectar. To carry back the pollen load to the hive, it is placed in baskets on their hind legs (*corbiculae*) [84]. To collect bee pollen for commercial uses, beekeepers use pollen traps at the entrance to the hive [85]. A great variability of the bee pollen’s appearance is caused by the differences in shape, color, size, and weight depending on the plant source [84].

Chemical analysis found about 200 compounds in bee pollen, including mean contents of: 30.8% carbohydrates, 22.7% protein, 10.4% essential amino acids, 5.1% lipids, 1.6% phenolic compounds, and 0.4% fatty acids. In addition, enzymes, coenzymes, vitamins (e.g., Vitamin A, B1, B2, B3, B5, B6, B9, B12, C, and E [85]), and small amounts of volatiles were detected [86].

Tu et al. [87] examined the effects of supplementing bee pollen to the diets of Holstein Frisian calves. Therefore, a total of 25 newborn female calves were randomly divided into five groups being fed a milk replacer to which was added: 0 (Control), 10 (10 BP), 25 (25 BP), 50 g of bee pollen/day (50 BP), or 5 g of bee pollen’s polysaccharides/day (5 PS). The animal research lasted 56 days. Growth performance, nutrient digestibility, and serum biochemical parameters were monitored during the testing period. It was found that the average daily gain was 20.4% (5 PS), 8.6% (10 BP), 13.2% (25 BP) and 6.3% (50 BP) greater in comparison to the control group [87]. Additionally, the apparent DM digestibility was increased by 9.7% (*p* = 0.007) or 8.2% (*p* = 0.019) for the 25 BP or 5 PS group [87]. For the serum biochemical parameters, there was no significant difference [87]. Besides these positives impacts on pre-ruminant calves, there are numerous studies claiming various other benefits for the supplementation of bee pollen in animal nutrition, e.g., improved fertility, positive effects on ovarian hormones, immunity or liver and kidney functions as reviewed by Abdelnour et al. [88].

## 11. Conclusions

Honey, beeswax, propolis, and royal jelly are natural products that are synthesized from collected pollen, resins, nectar, and honeydew. Although these products originally contribute to the honeybee’s nutrition—and, for beeswax, as a building material for the beehive—numerous studies on the health-promoting properties of honey, beeswax, propolis, royal jelly, and pollen have attracted attention regarding their use in animal nutrition. Various studies have shown positive effects on the performance of farmed animals following the use of apiculture by-products as feed additives.

Legal requirements for the use of bee products in animal nutrition are quite low, since there are not any legal definitions or specifications on the characteristics of honey, beeswax, propolis, royal jelly and pollen in the European feed law. However, apiculture by-products are listed in Regulation No. 68/2013 as feed stuffs, allowing their use in processed or natural form for animal nutrition.

In the future, further studies need to be carried out to fully understand which biochemical mechanisms are affected by the intake of apicultureby-products since the use of honey, beeswax, propolis, royal jelly, or pollen in the feeding of farmed animals may offer opportunities for minimizing the use of veterinary pharmaceuticals thanks to their health-promoting and performance-enhancing properties.

## Figures and Tables

**Table 1:** Overview of the protein concentrations of pollen from selected plants. Adapted from Pamminger et al. [27].

Genus	Number of Samples	Average Crude Protein in Pollen (Dry Mass) in %
*Acacia* MILL.	4	23.8
*Acer* L.	3	32.5
*Arctotheca* L.	3	18.4
*Brassica* L.	12	28.1
*Castanea* MILL.	3	22.9
*Centaurea* L.	5	23.5
*Cirsium* MILL.	4	23.9
*Cornus* L.	3	21.1
*Helianthus* L.	8	17.6
*Hypochaeris* L.	4	16.6
*Lupinus* L.	3	31.6
*Papaver* L.	3	25.0
*Prunus* L.	7	31.4
*Rubus* L.	4	23.8
*Salix* L.	5	27.5
*Sinapis* L.	5	26.4
*Solanum* L.	19	46.6
*Trifolium* L.	13	27.3
*Vicia* L.	5	29.4
*Zea* L.	8	17.8

## Data Availability

Not applicable.
